# YOLO-SCC: A Lightweight Object Detection Method for Mine Safety

**DOI:** 10.3390/s26154897

**Published:** 2026-08-03

**Authors:** Zhe Wu, Xuanrui Zhang, Yanping Cui, Yang Yang, Yingna Li

**Affiliations:** 1 School of Mechanical Engineering, Hebei University of Science and Technology, Shijiazhuang 050018, China; wuzhe@hebust.edu.cn (Z.W.); r18633005956@163.com (X.Z.); 2 Shijiazhuang Coal Mining Machinery Co., Ltd., Shijiazhuang 050031, China; freeyangya@163.com (Y.Y.); 15533113702@163.com (Y.L.)

**Keywords:** mine safety, underground personnel detection in coal mines, lightweight, attention mechanism, context aggregation

## Abstract

**Highlights:**

**What are the main findings?**

- Improved Precision and Localization Quality: Compared with YOLO26n, YOLO-SCC improves precision by 6.1 percentage points and mAP50-95 by 3.3 percentage points, while recall and mAP50 increase by 0.9 and 0.1 percentage points, respectively.
- Validation of Module Effectiveness: The designed SCC enhancement structure (SPDConv, CBAM, ContextAggregation) effectively addresses three core challenges in underground detection: detail loss, background interference, and insufficient contextual information. The efficacy of each module is validated through ablation studies.

**What are the implications of the main findings?**

- Provides a Feasible Solution for Lightweight Detection in Complex Industrial Scenarios: This study demonstrates that through carefully designed lightweight enhancement modules, the robustness of models in extreme environments can be significantly improved without substantially increasing computational costs. This offers important guidance for deploying high-performance safety monitoring systems on resource-constrained edge devices.
- Directly Advances Intelligent Safety in Coal Mining: By enabling more accurate and reliable detection of underground personnel, this method can help reduce safety incidents caused by missed detections or false alarms. It provides core technological support for developing a new generation of intelligent mine safety monitoring platforms and emergency response systems.

**Abstract:**

To address the challenges in personnel detection in underground coal mines, such as uneven lighting, dust occlusion, cluttered backgrounds, and significant scale variation of targets, this paper proposes a lightweight object detection method named YOLO-SCC. Based on the lightweight detection network YOLO26n, the SCC lightweight enhancement structure, composed of SPDConv, CBAM, and ContextAggregation, is constructed to improve feature representation and target recognition capability in complex underground environments while striving to maintain model compactness. Specifically, SPDConv optimizes the downsampling process to reduce the loss of feature details, which helps preserve edge and texture information of miners under low-light conditions, at long distances, or at small scales. CBAM adaptively weights features from both channel and spatial dimensions, enhancing the model’s focus on the main body of miners and suppressing irrelevant interference from light reflections, equipment structures, and dust noise. The ContextAggregation module aggregates richer contextual semantic information, strengthening the model’s discriminative ability for personnel targets under occlusion, dense distribution, and complex backgrounds. Experimental results show that with only a modest increase in parameters (from 2.38 MB to 2.88 MB) and computational cost (from 5.2 GFLOPs to 6.0 GFLOPs), YOLO-SCC achieves precision of 88.8%, recall of 82.2%, mAP50 of 85.3%, and mAP50-95 of 51.2%. These represent improvements of 6.1, 0.9, 0.1, and 3.3 percentage points, respectively, over the baseline YOLO26n. The results suggest that YOLO-SCC primarily improves false-positive suppression and localization quality in complex underground environments while maintaining a lightweight model scale.

## 1. Introduction

As a fundamental resource underpinning the global energy system and industrial production, coal’s strategic importance remains prominent. Its growing demand drives mining activities to greater depths, making the safety of underground personnel a core concern and a primary task for industry development [1,2,3,4]. The underground coal mine environment is complex, with frequent personnel movement, making operational safety a key issue in the intelligent construction of mines [5]. Accurate, real-time detection and identification of underground personnel not only provide fundamental support for personnel positioning, restricted area intrusion warnings, and operational behavior monitoring [6] but are also of great significance for improving mine safety management. However, underground mine environments typically suffer from insufficient lighting, localized strong reflections, dust diffusion, cluttered backgrounds, and target occlusion. These factors cause personnel targets in images to exhibit blurry edges, weak textures, and significant scale variations [7,8], posing considerable challenges to the stable application of object detection algorithms.

In recent years, the rapid development of deep learning has promoted the widespread application of object detection methods in complex industrial vision scenarios. Single-stage detection algorithms represented by the YOLO series, known for their fast detection speed, flexible deployment, and end-to-end training, have shown remarkable application prospects in fields such as remote sensing [9,10], agriculture [11,12], medicine [13,14], and industrial inspection [15,16]. For miner detection tasks in underground mines, the YOLO series offers a favorable balance between detection accuracy and real-time performance [17], making it a preferred choice. However, underground visual monitoring systems are often constrained by the computational power, storage resources, and power consumption of edge devices. Detection models must not only possess strong scene adaptability but also meet engineering requirements such as being lightweight, having a small parameter count, and having low computational complexity. Therefore, further improving detection performance in complex underground environments under the constraint of model scale has become a core challenge in this field.

To address the aforementioned challenges, this study adopts YOLO26n as the baseline model and proposes YOLO-SCC, a lightweight object detection method for underground personnel detection. Rather than simply stacking commonly used enhancement modules, YOLO-SCC is designed around three error sources that are particularly relevant to underground mine environments: the loss of fine-grained details during downsampling, false activations caused by complex backgrounds, and insufficient contextual representation under occlusion or dense target distributions.

Specifically, SPDConv is introduced at the early downsampling stage to preserve spatial details before feature compression, thereby mitigating the loss of edge and texture information for distant or small-scale personnel targets. CBAM is employed to selectively enhance personnel-related responses and suppress irrelevant activations caused by miner lamps, equipment structures, reflections, and dust. ContextAggregation is inserted after feature fusion in the neck to establish broader contextual dependencies and improve discrimination between personnel and visually similar background regions. By operating at different stages of the network, these modules form a progressive feature optimization pathway, from local detail preservation to target-focused feature refinement and, finally, contextual semantic aggregation.

Experimental results show that YOLO-SCC maintains a lightweight model scale, with 2.88M parameters and 6.0 GFLOPs. Compared with YOLO26n, YOLO-SCC improves precision by 6.1 percentage points and mAP50-95 by 3.3 percentage points, while recall and mAP50 increase by 0.9 and 0.1 percentage points, respectively. These results suggest that the proposed architecture primarily improves false-positive suppression and localization quality in complex underground environments while preserving the lightweight characteristics required for practical deployment.

## 2. Related Work

Jia P. et al. [18] proposed the YOLO-MD model, which integrates feature enhancement, multi-scale fusion, and feature calibration mechanisms. It effectively improves the accuracy and efficiency of personnel detection in complex underground environments, providing a real-time detection solution with higher recognition accuracy and reliability for mine safety monitoring.

Tian J. W. et al. [19] proposed a detection model based on Swin Transformer-YOLOv5. Under an edge-cloud collaborative architecture, it enhances the detection accuracy of mine video surveillance and its adaptability for edge deployment, offering a more efficient and reliable visual recognition solution for intelligent mine monitoring.

Li H. et al. [20] proposed a two-stage framework combining MCTE-GAN and YOLO-PKD. By enhancing low-light image quality and optimizing a lightweight detection model, it significantly improves the detection accuracy and real-time performance of mine monitoring under poor lighting conditions, providing a feasible intelligent perception solution for resource-constrained underground environments.

Zhang L. et al. [21] proposed the EAW-YOLO model, which achieves accurate real-time identification of personnel and vehicle targets underground by embedding an improved YOLOv11. Coupled with a PLC system for controlling air doors, it significantly enhances the automation level and safety reliability of mine ventilation management and control.

Zhang L. et al. [22] proposed the DBE-YOLO model. Through structural improvements, it enhances the accuracy and speed of detecting underground protective equipment, enabling reliable monitoring of safety gear in complex environments. This provides an efficient real-time visual solution for on-site safety management in coal mines.

Zhang F. et al. [23] proposed the DrillNet model, with an improved YOLO-GC network at its core. By integrating multi-scale feature fusion and global context aggregation, it achieves high-precision, real-time directional detection and automatic counting of drill rods in complex mine environments, significantly elevating the intelligent monitoring level of drilling operations.

Yu X. [24], Zhang Z. [25], Fan Y. [26], Wang Z. [27], Xin F. [28], and Jin H. et al. [29], based on the YOLOv8 model, respectively targeted key scenarios in coal mines such as foreign object monitoring on conveyor belts, personnel safety behavior recognition, hazardous area intrusion warning, equipment status perception, and coal sorting optimization. Through algorithmic improvements, they achieved more accurate, faster, and lighter visual detection capabilities in complex underground environments, collectively advancing the closed-loop application of mine intelligence from perception to control.

Li X. et al. [30] proposed a Faster R-CNN-LSTM dual-stream network, which significantly improves action recognition accuracy through temporal and spatial feature fusion. However, its high computational complexity may make it less suitable than lightweight YOLO-series models for mine monitoring scenarios requiring high real-time response.

Zhai S. et al. [31] proposed the DF-SSD model. By introducing a DenseNet backbone and multi-scale feature fusion, it significantly enhances feature extraction capability and small object detection performance, achieving better parameter efficiency than the original SSD and Faster R-CNN. However, the computational burden introduced by its densely connected structure poses a significant challenge under the harsh deployment constraints of high real-time demand and low computing power underground. Its practical efficiency may not match that of lightweight YOLO models specifically optimized for similar scenarios.

Du S. et al. [32] proposed TSD-YOLO, an improved YOLOv8-based traffic sign detection method. They introduced an SPD module to enhance multi-scale feature representation and a Select Kernel attention mechanism to reduce background interference. Experiments showed improved mAP50 while maintaining high detection speed.

Li N. et al. [33] proposed IHENet for low-light object detection. The method employs an illumination-robust feature extractor and a hierarchical feature enhancement network to improve illumination adaptation and preserve detection-relevant details. Experiments showed improved mAP50 with reduced model parameters.

## 3. YOLO-SCC Lightweight Object Detection Method

**Figure 1:**
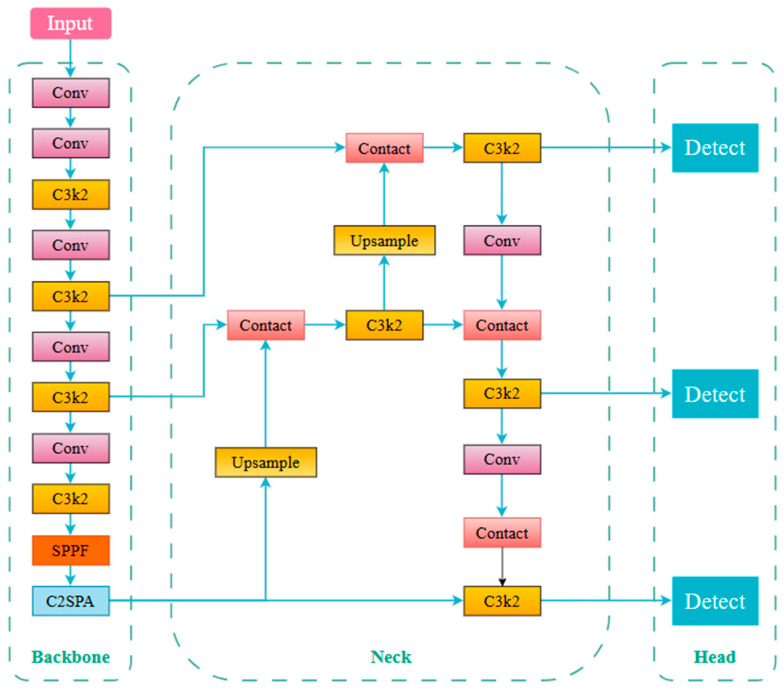
Architecture of the YOLO26n model.

In response to the core challenges of detail loss, background interference, and semantic ambiguity in underground personnel detection, this study proposes YOLO-SCC, an enhanced lightweight object detection model based on YOLO26n [34]. The original architecture of YOLO26n is shown in Figure 1.

YOLO-SCC is designed as a progressive feature optimization framework that aims to preserve local details, suppress background interference, and enhance contextual semantic representation while maintaining a lightweight model scale. Its SCC enhancement structure consists of three module types: SPDConv, CBAM, and ContextAggregation. Among them, CBAM is deployed at two different stages of the network to perform feature refinement for different purposes.

Specifically, SPDConv is introduced in the early downsampling layers of the backbone to reduce the loss of fine-grained spatial details. A CBAM module is placed in the middle layers of the backbone to suppress background interference. ContextAggregation is inserted after feature fusion in the neck to enhance contextual discrimination, and an additional CBAM module is applied before generating shallow output features to further refine detail-rich but noise-sensitive features. As summarized in Table 1, these components are deployed at different stages of the network and perform complementary functions, thereby forming a progressive feature optimization pathway for underground personnel detection.

From a feature flow perspective, the SCC structure is not intended as a simple serial combination of independent modules. Its deployment follows the order in which three types of feature errors arise in underground coal mine imagery and feature extraction: local detail loss caused by early downsampling; background-induced false activations caused by miner lamps, reflections, dust, and equipment structures; and contextual ambiguity caused by occlusion or dense target distributions. SPDConv is therefore placed before substantial feature compression to preserve local edge and texture cues. These detail-preserved features provide a more reliable basis for the subsequent CBAM to distinguish personnel-related responses from visually similar background activations. By suppressing irrelevant activations before feature fusion, CBAM also provides cleaner inputs for ContextAggregation. ContextAggregation then exploits the relationship between a candidate region and its surrounding context to improve discrimination for partially occluded or densely distributed personnel targets. The additional CBAM module in the neck further refines shallow, detail-rich output features after feature fusion.

This ordering creates functional complementarity rather than simple module accumulation. When SPDConv is used alone, it may preserve both personnel details and background clutter. When CBAM is used without sufficient detail preservation, it may attenuate weak responses from distant, small-scale, or heavily occluded personnel targets. Similarly, ContextAggregation cannot recover local evidence that has already been lost during downsampling, nor can it fully eliminate background interference propagated from earlier stages. In the complete SCC structure, detail preservation supports attention-based feature selection, attention filtering improves the reliability of contextual aggregation, and contextual aggregation resolves ambiguities that remain after local feature refinement. Therefore, the SCC structure constitutes a task-oriented feature-flow mechanism tailored to underground personnel detection, rather than an arbitrary stacking of existing modules.

### 3.1. Feature Enhancement and Focusing in the Backbone Network

At the source of feature extraction, this study introduces the SPDConv module to replace two critical downsampling layers in the original network. This method stems from the concept of space-to-depth image transformation, aiming to substitute the information-losing pooling or strided convolution operations with lossless feature reorganization.

Given an intermediate feature map $X\in {\mathbb{R}}^{S\times S\times C_{1}}$, where ℝ is the standard symbol for the set of real numbers, the SPD layer first systematically slices it along the spatial dimension into a sequence of $scale^{2}$ sub-feature maps. This module selects scale=2. These sub-feature maps $f_{x,y}$ are generated by the following slicing operation:

(1) $$\begin{array}{l} f_{0,0}=X[0:S:\mathrm{scale},0:S:\mathrm{scale}]\ldots f_{\mathrm{scale}-1,0}=\\ X[\mathrm{scale}-1:S:\mathrm{scale},0:S:\mathrm{scale}]; \end{array}$$

(2) $$\begin{array}{l} f_{0,1}=X[0:S:\mathrm{scale},1:S:\mathrm{scale}]\ldots f_{\mathrm{scale}-1,1}=\\ X[\mathrm{scale}-1:S:\mathrm{scale},1:S:\mathrm{scale}]; \end{array}$$

(3) $$\begin{array}{l} f_{0,\mathrm{scale}-1}=X[0:S:\mathrm{scale},\mathrm{scale}-1:S:\mathrm{scale}]\ldots f_{1,\mathrm{scale}-1}\ldots \\ f_{\mathrm{scale}-1,\mathrm{scale}-1}=X[\mathrm{scale}-1:S:\mathrm{scale},\mathrm{scale}-1:S:\mathrm{scale}]. \end{array}$$

Each sub-feature map $f_{x,y}$ has a size of $\left(S/scale\right)\times \left(S/scale\right)\times C_{1}$. Subsequently, these sub-feature maps are concatenated along the channel dimension to obtain the reorganized feature map X′:

(4) $$X^{\prime }=\mathrm{Concat}\left(f_{0,0},f_{1,0},\ldots ,f_{\mathrm{scale}-1,\mathrm{scale}-1}\right)\in {\mathbb{R}}^{\frac{S}{\mathrm{scale}}\times \frac{S}{\mathrm{scale}}\times ({\mathrm{scale}}^{2}\cdot C_{1})}$$

**Figure 2:**
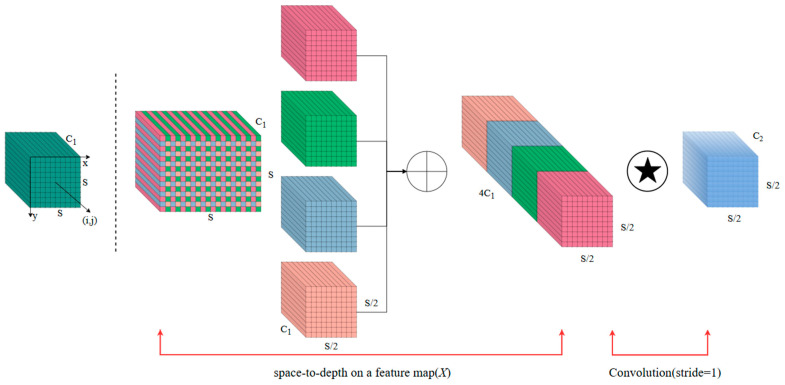
Schematic diagram of the SPDConv structure (*scale* = 2).

The spatial dimensions of the feature map are compressed from S×S to $\left(S/scale\right)\times \left(S/scale\right)$, while the number of channels is expanded by a factor of $scale^{2}$. Subsequently, a convolutional layer with a stride of 1 performs channel fusion and adjustment on $X^{\prime }$, outputting the feature map $X^{\prime \prime }$ with the target channel number $C_{2}$ [35]. Figure 2 below illustrates the SPDConv operation when scale=2 is selected, where the symbol ⊕ represents channel concatenation of the four sub-feature maps generated by space-to-depth transformation.

(5) $$X^{\prime }\left(\frac{S}{scale},\frac{S}{scale},scale^{2}C_{1}\right)\overset{\mathrm{Conv}\;(\mathrm{stride}=1)}{\to }X^{\prime \prime }\left(\frac{S}{scale},\frac{S}{scale},C_{2}\right)$$

The entire SPDConv operation, through the aforementioned mathematically strict information-preserving spatial reorganization of feature maps, replaces the information-discarding pooling or strided convolution in traditional downsampling. This maximizes the retention of edge and texture details for small-scale personnel targets while compressing the feature map size, thereby effectively mitigating the issue of detail loss caused by resolution reduction at the fundamental level.

**Figure 3:**
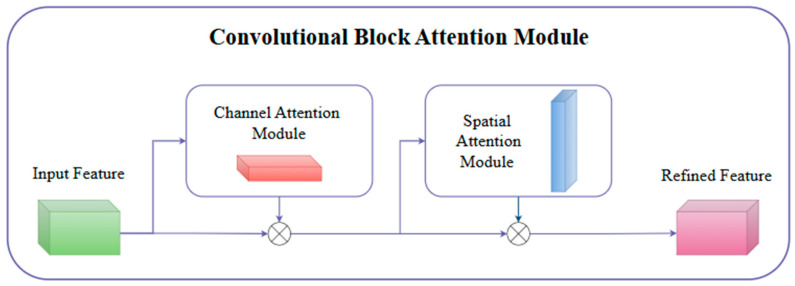
Schematic diagram of the CBAM structure. Where the cross symbol ⊗ denotes element-wise multiplication between the original feature map and attention weights.

Subsequently, the CBAM is embedded into the middle layers of the backbone network. Its structure is illustrated in Figure 3, where the cross symbol ⊗ denotes element-wise multiplication between the original feature map and attention weights. The module sequentially infers attention maps along both the channel and spatial dimensions, enabling the adaptive refinement of the input features [36].

Given an input feature map $F\in {\mathbb{R}}^{C\times H\times W}$, CBAM first computes a one-dimensional channel attention map $M_{C}\in {\mathbb{R}}^{C\times 1\times 1}$ by applying a shared multilayer perceptron (MLP) to both the average-pooled and max-pooled features and then summing the results:

(6) $$\begin{array}{l} M_{c}(F)=\sigma \left(\mathrm{MLP}\left(\mathrm{AvgPool}(F)\right)+\mathrm{MLP}\left(\mathrm{MaxPool}(F)\right)\right)=\\ \sigma \left(W_{1}\left(W_{0}\left(F_{\mathrm{avg}}^{c}\right)\right)+W_{1}\left(W_{0}\left(F_{\max}^{c}\right)\right)\right) \end{array}$$

**Figure 4:**
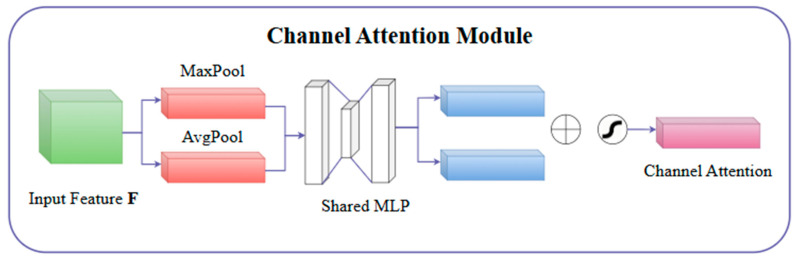
Structure of the channel attention module.

In Equation (6): σ denotes the Sigmoid activation function, $W_{0}\in {\mathbb{R}}^{C/r\times C}$ and $W_{1}\in {\mathbb{R}}^{C\times C/r}$ are the shared MLP weights, and r is the reduction ratio. The channel-refined feature $F^{\prime }$ is obtained through element-wise multiplication, as illustrated in Figure 4, where ⊕ denotes element-wise addition of two feature vectors, and σ represents the Sigmoid activation function shown by the circular curved line icon in the diagram.

(7) $$F^{\prime }=M_{c}(F)⊗F$$

In Equation (7), ⊗ denotes the element-wise multiplication.

**Figure 5:**
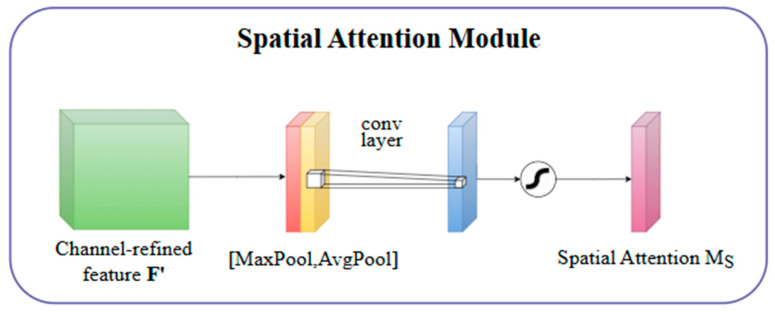
Structure of the spatial attention module.

Subsequently, a two-dimensional spatial attention map $M_{s}\in {\mathbb{R}}^{1\times H\times W}$ is computed based on $F^{\prime }$. This module performs average pooling and max pooling separately along the channel axis. The resulting two two-dimensional descriptors are then concatenated, and the final weights are generated by a convolutional layer, as shown in Figure 5. In this figure, cubic shapes denote feature tensors, while colored prismatic blocks represent intermediate feature maps. The Sigmoid activation function is applied to generate the final spatial attention map $M_{S}$.

(8) $$\begin{array}{l} M_{s}(F^{\prime })=\sigma \left(f^{7\times 7}\left(\left[\mathrm{AvgPool}(F^{\prime });\mathrm{MaxPool}(F^{\prime })\right]\right)\right)=\\ \sigma \left(f^{7\times 7}\left(\left[F_{avg}^{\prime s};F_{\max}^{\prime s}\right]\right)\right) \end{array}$$

The final output feature F″ is

(9) $$F^{\prime \prime }=M_{s}(F^{\prime })⊗F^{\prime }$$

Through the aforementioned module, the network is able to adaptively calibrate channel and spatial responses during the early stages of feature extraction. This allows it to initially focus on the main body region of personnel and suppress strong background interferences in underground mines, such as miners’ lamps, equipment, and dust.

### 3.2. Progressive Multi-Scale Optimization in the Neck Network

**Figure 6:**
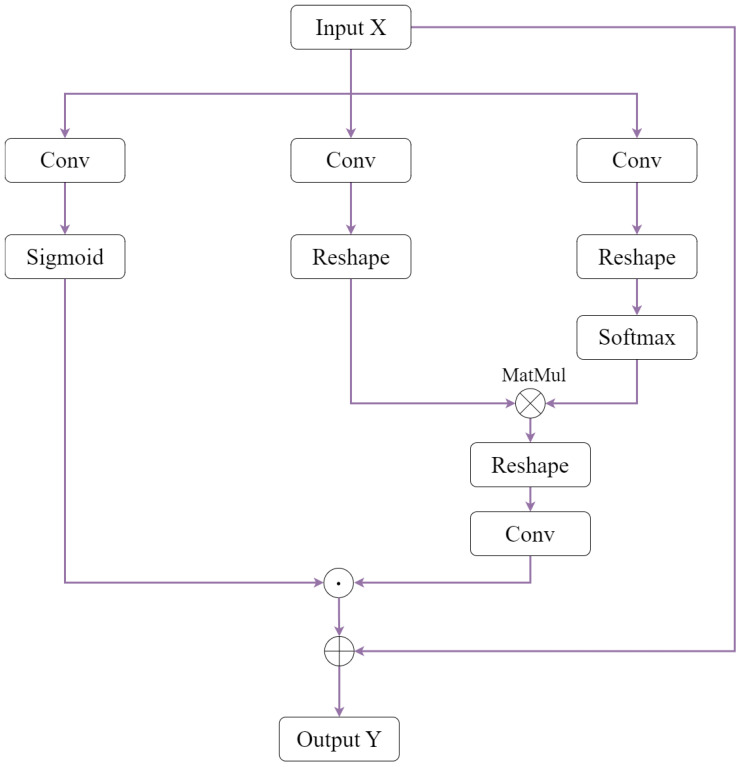
Architecture of the ContextAggregation module.

The ContextAggregation module is introduced after feature fusion in the neck to aggregate global contextual information and redistribute it according to the responses at different spatial positions, as illustrated in Figure 6, In this figure, ⊗ denotes matrix multiplication, ⊙ denotes element-wise multiplication, with Sigmoid and SoftMax are activation functions. Inspired by the general concept of contextual aggregation [37], the implemented module uses attention-based global context pooling and spatial gating to enhance feature representations.

Given an input feature map $X\in {\mathbb{R}}^{B\times C\times H\times W}$, where B, C, H, and W denote the batch size, number of channels, height, and width, respectively, let L=HW denote the total number of spatial positions. The intermediate channel dimension is defined as C′=max(C/r,1), where r is the channel reduction ratio.

First, a spatial gating map is generated using a 1×1 convolution followed by a Sigmoid activation function:

(10) $$A_{s}=\sigma \left(f_{a}(X)\right)$$

where $A_{s}\in {\mathbb{R}}^{B\times 1\times H\times W}$, $f_{a}(∙)$ denotes a 1×1 convolution, and $\sigma \left(∙\right)$ denotes the Sigmoid function.

Meanwhile, another 1×1 convolution is used to generate the spatial context weights. The spatial dimensions are flattened, and Softmax normalization is performed over all L spatial positions:

(11) $$K={\mathrm{Softmax}}_{L}\left(\mathrm{Reshape}\left(f_{k}(X)\right)\right)$$

where $K\in {\mathbb{R}}^{B\times 1\times L\times 1}$, and the weights over all spatial positions sum to one. $f_{k}(∙)$ denotes a learnable 1×1 convolution that maps the input channels from C to 1, $\mathrm{Reshape}\left(∙\right)$ denotes a tensor-dimension rearrangement operation, ${\mathrm{Softmax}}_{L}\left(∙\right)$ denotes Softmax normalization along the L spatial positions, and K denotes the normalized spatial context weights.

The value features are generated through a separate 1×1 convolution and reshaped as

(12) $$V=\mathrm{Reshape}\left(f_{v}(X)\right)$$

where $V\in {\mathbb{R}}^{B\times 1\times C^{\prime }\times L}$.

The global context feature is obtained by performing weighted aggregation over all spatial positions:

(13) $$G=\mathrm{Reshape}(VK)=\sum_{i=1}^{L}K_{i}V_{i},$$

where $G\in {\mathbb{R}}^{B\times C^{\prime }\times 1\times 1}$. This operation summarizes information from the entire feature map according to the learned spatial context weights. The aggregated context feature is then projected back to the original channel dimension and modulated by the spatial gating map. Finally, a residual connection is applied:

(14) $$Y=X+f_{m}(G)⊙A_{s}$$

where $f_{m}(∙)$ denotes a 1×1 convolution that maps the channel dimension from C′ to C and ⊙ denotes element-wise multiplication with broadcasting. The output $Y\in {\mathbb{R}}^{B\times C\times H\times W}$ preserves the original input features while selectively enhancing spatially relevant regions with globally aggregated contextual information.

Furthermore, before generating the final shallow-level feature maps used for detecting small targets, a CBAM module is reintroduced. This module performs a secondary refinement of the shallow features, which are rich in detail but also contain more noise, in both spatial and channel dimensions. This ensures the accuracy of the model’s focus on distant or occluded targets.

**Figure 7:**
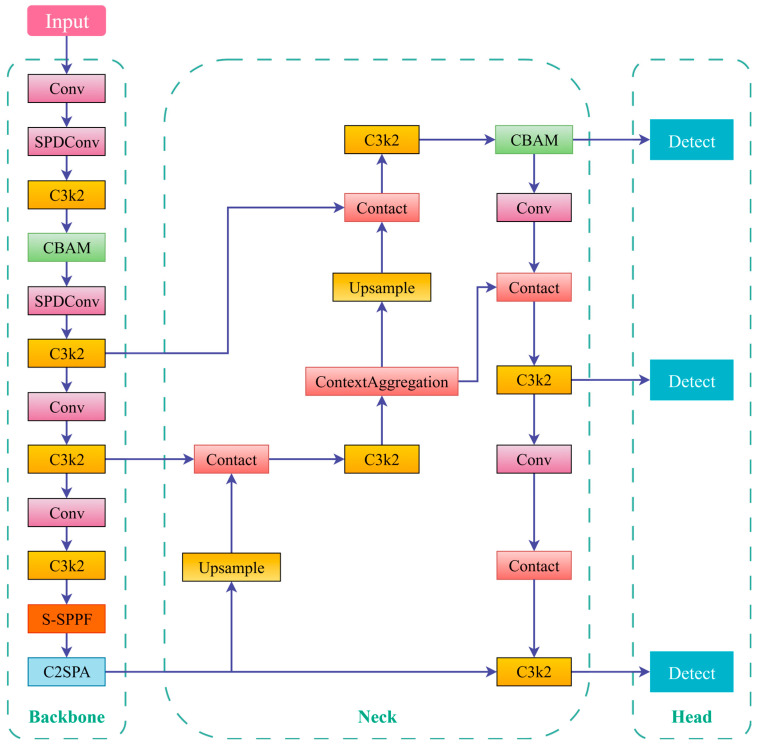
Architecture of the YOLO-SCC model (S-SPPF denotes the simplified SPPF configuration adopted to control model complexity).

This design establishes a clear progressive feature optimization pathway. The overall architecture of YOLO-SCC is illustrated in Figure 7 below. The input image is processed by the backbone network, which generates multi-scale foundational features. This is achieved through SPDConv-guaranteed preservation of low-level details and preliminary CBAM-guided feature focusing. These features then undergo upsampling, downsampling, and fusion within the neck network. ContextAggregation is applied to consolidate and integrate high-level semantics, and a final CBAM performs precise calibration of the output features. Finally, the optimized feature maps at three different scales are fed into the detection head to predict personnel targets of large, medium, and small scales, respectively. The entire process systematically enhances the model’s capabilities—from local detail perception to global semantic consistency—under the constraint of maintaining a lightweight architecture.

## 4. Experiments and Results

### 4.1. Parameter Configuration and Dataset

To comprehensively evaluate the model’s performance in complex real-world underground mining environments, this study developed a dedicated dataset for mine personnel detection. The dataset consists of two components: the publicly available coal_miner dataset sourced from the RoboFlow platform, and the widely used multi-scenario underground dataset DsLMF+. Through screening and merging of the two, a final dataset comprising 3629 real underground images was constructed. To ensure the objectivity of the evaluation, all images were randomly divided into training, validation, and test sets in a 7:1.5:1.5 ratio.

The experimental dataset was constructed by combining the publicly available coal_miner dataset from Roboflow with a subset adapted from DsLMF+ for underground personnel detection. The final dataset contains 3629 images and 7050 annotated personnel instances. The dataset includes a single detection category, namely, person (class 0). According to the original dataset identifiers provided by the data sources, no overlapping records were identified between the two source datasets. The training, validation, and test sets contain 2538, 541, and 550 images, with 5277, 919, and 854 annotated instances, respectively. The widths and heights of the original images range from 588 to 2592 pixels and from 480 to 1944 pixels, respectively.

The annotations provided by the original public datasets were retained, and no additional manual relabeling was performed by the authors. To assess annotation file integrity, an automated check of the YOLO labels was conducted. No missing label files, empty label files, malformed annotation entries, or unreadable images were found. The detailed dataset statistics are summarized in Table 2.

**Figure 8:**
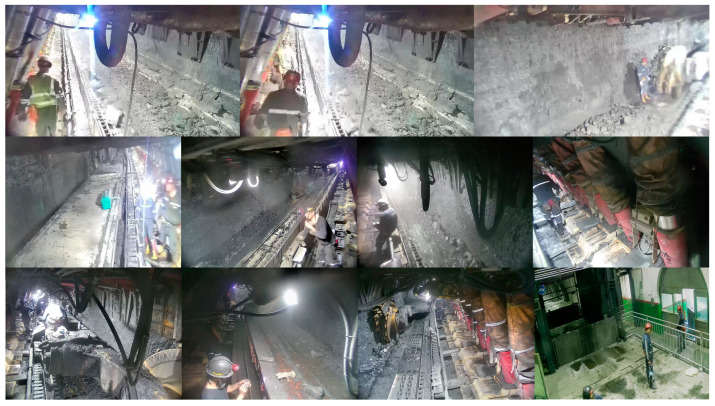
Coal mine miner dataset.

The images in the dataset cover multiple typical operational scenarios in underground mines, including tunneling, support installation, transportation, and maintenance, as shown in Figure 8 below. Personnel targets encompass various postures such as standing, walking, and crouching. The lighting conditions in the scenes are complex and variable, presenting challenges such as localized strong lighting, backlighting, low illumination, and dust interference, which authentically reflect the visual detection difficulties in coal mine environments. All personnel targets are accurately annotated with bounding boxes. The construction of this dataset aims to provide a representative and comprehensively covered benchmark for research on lightweight object detection in underground mines.

The key hyperparameter settings used for model training and evaluation are listed in Table 3 below and the computer hardware and software configurations on which the experiments rely are detailed in Table 4 below.

### 4.2. Model Evaluation Metrics

To comprehensively evaluate model performance, this study adopts a commonly used evaluation system in the field of object detection, which mainly includes the following metrics:

(1) Precision (P): Precision is a core metric for assessing model quality, measuring the proportion of true positive samples among all predicted positive samples. It reflects the model’s ability to identify correct detections, where a high precision means the identified targets are mostly correct, with fewer false positives.
(2) Recall (R): Recall measures the proportion of all actual positive samples that are successfully detected by the model, reflecting the model’s ability to find all relevant targets. A high recall indicates fewer missed detections.
(3) Mean Average Precision at IoU = 0.5 (mAP50): This metric reflects the model’s overall performance under a relaxed localization criterion (Intersection over Union, IoU ≥ 0.5).
(4) Mean Average Precision over IoU Thresholds 0.5 to 0.95 (mAP50-95): This metric is considered the gold standard for evaluating the spatial accuracy between predicted bounding boxes and ground truth boxes. It comprehensively assesses the model’s localization precision across varying IoU thresholds. For safety-critical applications in mines, such as personnel positioning and distance estimation, mAP50-95 holds extremely high reference value.
(5) Parameter Count (Params): The total number of all learnable parameters in the model, measured in millions (M). It directly relates to the model’s size and affects storage and loading requirements.
(6) Computational Complexity (GFLOPs): The number of floating-point operations required for a single forward inference of the model, measured in billions. This metric is a key indicator of the model’s computational complexity.

The formula for precision is

(15) $$\mathrm{Precision}=\frac{TP}{TP+FP}$$

The formula for recall is

(16) $$\mathrm{Recall}=\frac{TP}{TP+FN}$$

The formula for average precision is

(17) $$mAP=\frac{1}{m}\sum_{i=1}^{m}AP_{i}$$

### 4.3. Ablation Study

To scientifically validate the effectiveness of each module in the proposed YOLO-SCC structure and their synergistic interactions, an ablation study on the SCC structure was conducted. Using YOLO26n as the baseline model, a control variable approach was employed to progressively integrate each enhancement module, resulting in five comparative model configurations. This design aims to investigate the individual contribution and combined effects of each module on the final detection performance. All comparative experiments were conducted under the same dataset and experimental environment described in Section 4.1. The models were rigorously evaluated using the metrics defined in Section 4.2.

Table 5 summarizes the ablation results of the proposed SCC enhancement structure. YOLO26n provides a compact baseline with a precision of 0.827, recall of 0.813, mAP50 of 0.852, and mAP50-95 of 0.479. The results of the single-module configurations indicate that the three modules affect different aspects of feature representation and introduce different performance trade-offs when used independently. To control model complexity, all ablation variants except the original YOLO26n baseline employ a simplified SPPF configuration, denoted as S-SPPF. The S-SPPF module alone has a negligible effect on the detection performance, and the performance variations observed in the ablation experiments mainly result from the introduced feature enhancement modules rather than the SPPF configuration. In the CBAM-only and ContextAggregation-only variants, the parameter reduction achieved by S-SPPF exceeds the additional parameters introduced by the corresponding module, resulting in slightly lower parameter counts than the baseline.

When ContextAggregation is introduced alone, precision increases from 0.827 to 0.909, while mAP50 and mAP50-95 increase slightly to 0.856 and 0.481, respectively. However, recall decreases from 0.813 to 0.781. Similarly, the CBAM-only configuration improves precision to 0.906 and mAP50 to 0.857, but recall decreases to 0.794 and mAP50-95 changes only slightly to 0.480. These results suggest that ContextAggregation and CBAM can enhance the discrimination of personnel-related features and suppress some background-induced false activations, but when used alone, they may also suppress weak responses associated with difficult personnel targets, thereby reducing detection coverage.

SPDConv alone achieves a precision of 0.904, but its recall decreases substantially to 0.744, while mAP50 and mAP50-95 decrease to 0.823 and 0.462, respectively. This result does not imply that SPDConv is ineffective for preserving local details. Rather, it indicates that local detail preservation alone is insufficient to ensure stable detection coverage in complex underground scenes. Without subsequent feature selection and contextual discrimination, the detail-preserved features may still contain substantial background clutter or may not provide sufficient semantic information for reliably identifying difficult personnel targets.

The two-module configurations further demonstrate the complementary roles of the proposed components. Compared with SPDConv alone, the combination of SPDConv and ContextAggregation increases recall from 0.744 to 0.807 and improves mAP50-95 from 0.462 to 0.497, while restoring mAP50 to 0.852. This result indicates that ContextAggregation can compensate for part of the limitation of SPDConv by incorporating broader contextual information and improving the semantic discrimination of detail-preserved features. In contrast, the SPDConv + CBAM configuration achieves a precision of 0.854, recall of 0.747, mAP50 of 0.820, and mAP50-95 of 0.482. Although its mAP50-95 is slightly higher than that of SPDConv alone, the configuration does not provide a balanced improvement in detection coverage and localization quality. The combination of ContextAggregation and CBAM achieves a precision of 0.831, recall of 0.785, mAP50 of 0.845, and mAP50-95 of 0.490. Although this configuration improves mAP50-95 compared with the baseline model, it does not achieve a favorable balance across all detection metrics and has the highest computational cost among the evaluated variants, with 2.957744M parameters and 7.3 GFLOPs. Therefore, simply combining attention-based refinement and contextual aggregation does not necessarily produce the most effective architecture.

Among all configurations, YOLO-SCC achieves the highest recall of 0.822 and the highest mAP50-95 of 0.512. Compared with YOLO26n, YOLO-SCC improves precision by 6.1 percentage points, recall by 0.9 percentage points, mAP50 by 0.1 percentage points, and mAP50-95 by 3.3 percentage points. These results indicate that the complete SCC structure provides a more balanced trade-off between detection coverage, localization quality, and model complexity than the individual modules or partial combinations. Specifically, SPDConv preserves local edge and texture cues during early downsampling; CBAM suppresses irrelevant activations caused by miners’ lamps, equipment structures, reflections, and dust; and ContextAggregation integrates broader contextual information to distinguish personnel from visually similar background regions. When these components are deployed at their designated stages, local detail preservation supports feature selection, attention-based filtering improves the reliability of contextual aggregation, and contextual information further resolves ambiguities that remain after local feature refinement.

### 4.4. Comparative Experiment

The inference speed was calculated using the average per-image processing time reported during test set evaluation. The total processing latency consists of preprocessing, model inference, and post-processing time, while the loss calculation time was excluded. Accordingly, FPS was calculated as

(18) $$T_{\mathrm{total}}=T_{\mathrm{preprocess}}+T_{\mathrm{inference}}+T_{\mathrm{postprocess}}$$

(19) $$\mathrm{FPS}=\frac{1000}{T_{\mathrm{total}}}$$

where $T_{\mathrm{total}}$ is expressed in milliseconds per image.

Table 6 compares YOLO-SCC with conventional detectors, a Transformer-based detector, a mine-specific detector, and several lightweight YOLO variants. Faster R-CNN achieves the highest recall of 0.897 and mAP50 of 0.857. However, its 137.099M parameters, 370.21 GFLOPs, and processing speed of 17.5 FPS indicate a relatively high runtime cost. RT-DETR-R50 achieves a precision of 0.830 and a processing speed of 44.84 FPS, but its computational requirements remain considerably higher than those of the lightweight YOLO models [38]. Rep-YOLO, which was specifically developed for mine-personnel detection, achieves the highest mAP50 of 0.862. Nevertheless, its 23.067M parameters, 68.9 GFLOPs, and processing speed of 60.24 FPS indicate substantially higher computational costs than the nano-scale YOLO variants [39]. SSD achieves the highest precision of 0.9181, but its recall is limited to 0.7084, with a processing speed of 61.8 FPS.

Among the compared lightweight YOLO models, YOLOv8n achieves the highest processing speed of 218.45 FPS and the highest recall of 0.840. YOLOv11n and YOLOv12n contain fewer parameters than YOLO-SCC, with 2.582M and 2.557M parameters, respectively. Meanwhile, YOLO26n has the lowest parameter count and GFLOPs among the evaluated YOLO variants, with 2.375M parameters and 5.2 GFLOPs. These results indicate that no single lightweight model consistently dominates all detection accuracy, model complexity, and processing speed metrics.

YOLO-SCC achieves the highest mAP50-95 of 0.512 among all evaluated models and the highest precision of 0.888 among the compared YOLO variants. It also obtains a recall of 0.822 and mAP50 of 0.853. With 2.883M parameters, 6.0 GFLOPs, and a processing speed of 192.31 FPS, YOLO-SCC maintains a compact model scale and real-time processing capability under the stated evaluation conditions. It should be noted that GFLOPs and FPS do not exhibit a simple linear relationship, as inference speed is also affected by factors such as network architecture, operator implementation, and hardware execution efficiency. Therefore, the slightly higher FPS of YOLO-SCC compared with YOLO26n, despite its higher GFLOPs, results from the combined effects of these factors rather than the computational complexity alone.

Although YOLO-SCC does not achieve the highest value in every individual metric, it was selected as the final architecture because it provides a favorable combination of detection accuracy, model complexity, and processing speed. In particular, the improvement in mAP50-95 indicates better overall detection and localization performance across different IoU thresholds, while the compact parameter count, limited computational cost, and high FPS preserve its suitability for real-time mine personnel monitoring. Therefore, YOLO-SCC offers a practical trade-off that is consistent with the accuracy and deployment requirements of complex underground environments.

### 4.5. Visualization and Analysis of Results

**Figure 9:**
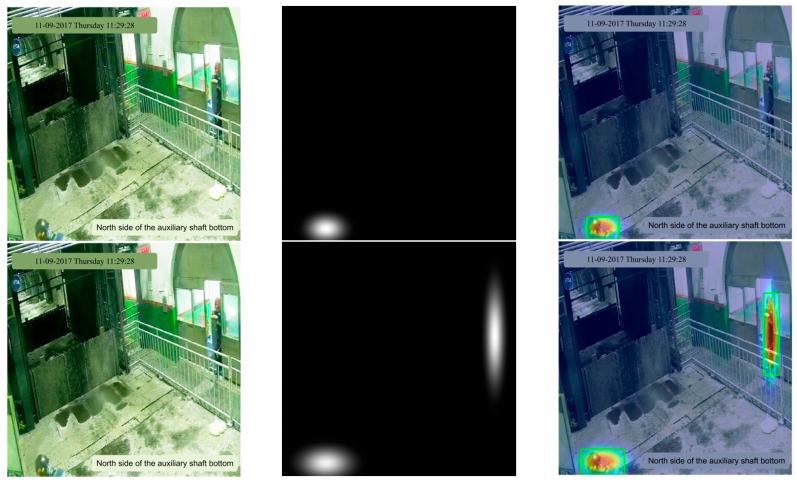
**Scene 1**: YOLO26n model recognition results. SCC model recognition results.

**Figure 10:**
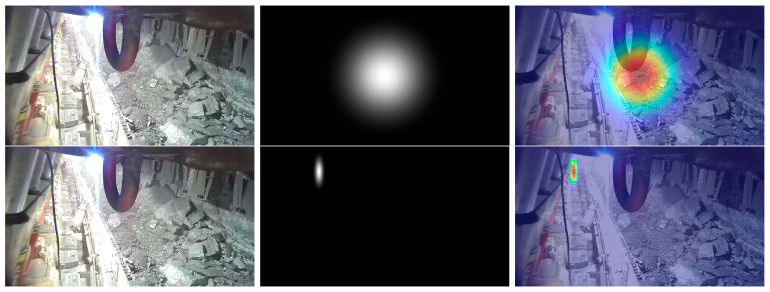
**Scene 2**: YOLO26n model recognition results. SCC model recognition results.

**Figure 11:**
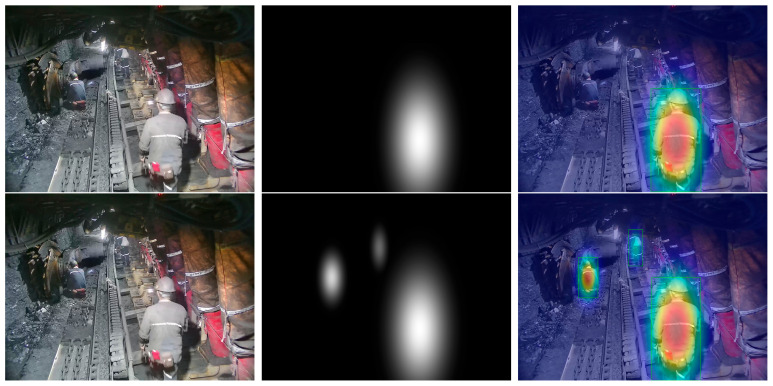
**Scene 3**: YOLO26n model recognition results. SCC model recognition results.

**Figure 12:**
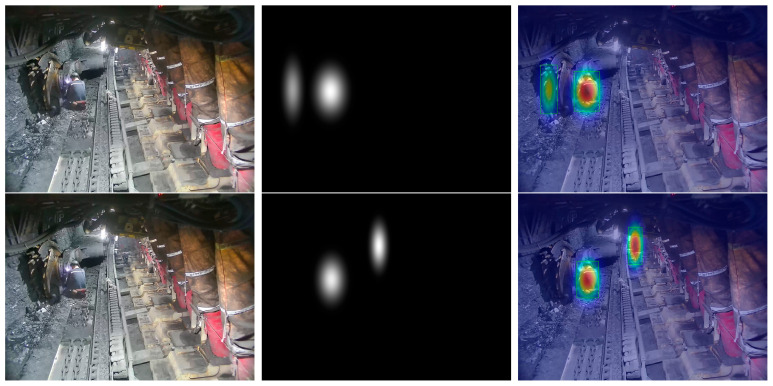
**Scene 4**: YOLO26n model recognition results. SCC model recognition results.

**Figure 13:**
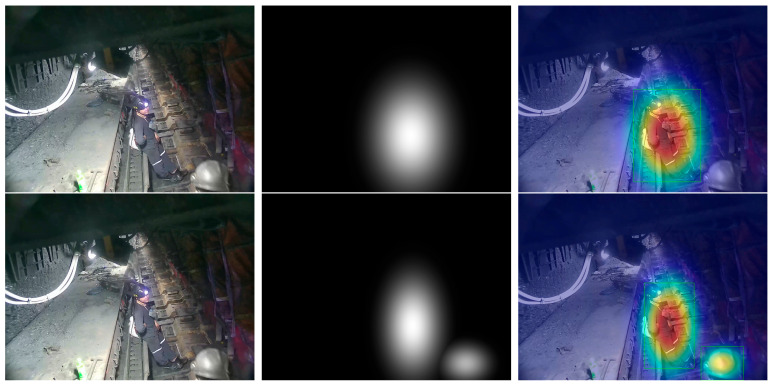
**Scene 5**: YOLO26n model recognition results. SCC model recognition results.

This section presents a comparative visual analysis of the feature attention patterns between the baseline model YOLO26n and the improved model YOLO-SCC in specific underground scenarios characterized by strong lighting, dust, and occlusion. As shown in the comparative heatmaps in Figure 9, Figure 10, Figure 11, Figure 12 and Figure 13, a higher color temperature indicates a stronger focus and reliance of the model on the corresponding region. The analysis reveals that under the same interference conditions, the YOLO-SCC model’s high-activation regions are more concentrated and stably focused on the key discriminative features of personnel targets, while maintaining lower activation responses to background and noise interference. In contrast, the feature attention regions of the YOLO26n model exhibit a certain degree of dispersion in similar scenarios and are more prone to activation in non-target areas or display positional deviations in activation under strong light or dust interference. These results, from an interpretability perspective, indicate that the introduced SCC structure helps enhance the model’s feature selectivity and anti-interference capability in complex underground environments, making its decision-making process more focused on regions highly relevant to the target semantics.

### 4.6. Failure Case Analysis and Limitations

**Figure 14:**
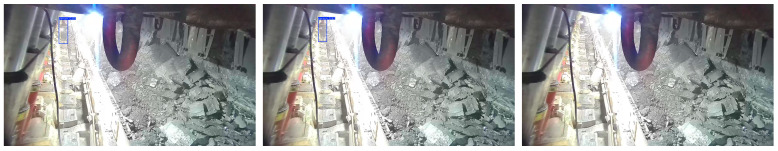
Strong illumination interference.

**Figure 15:**
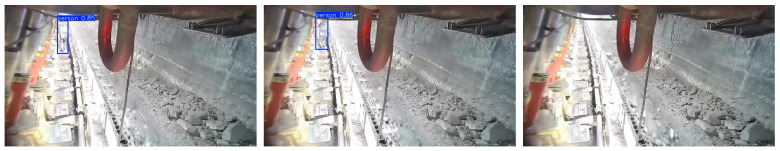
Extremely small target and dust interference.

**Figure 16:**
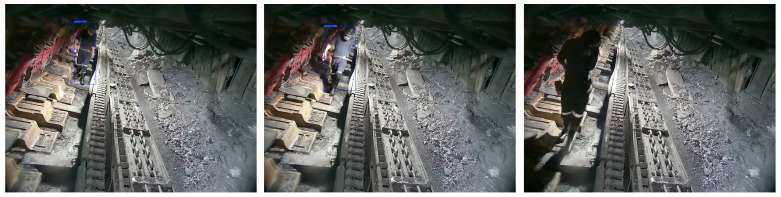
Insufficient illumination and low contrast.

Figure 14, Figure 15 and Figure 16 show representative failure cases of YOLO-SCC under challenging underground conditions. Figure 14 illustrates missed detections caused by strong illumination interference. In this scenario, YOLO-SCC can detect the personnel target in some images, but when local overexposure weakens the visible personnel features, the model may fail to detect the target. Figure 15 shows missed detections caused by extremely small targets and dust interference. When the personnel occupy only a very small region of the image and dust further weakens the available texture and contour information, the model may fail to generate a reliable detection response. Figure 16 presents missed detections under insufficient illumination and low target-background contrast. The blue boxes represent detected targets and their corresponding confidence scores. In this case, the visual response of the personnel region becomes weak, and the personnel target may be confused with the surrounding roadway background, resulting in missed detection. In each figure, the first two images show detectable cases under similar conditions, while the third image shows a missed detection case.

## 5. Conclusions

This study addressed the challenges of personnel detection in complex underground coal mine environments by proposing YOLO-SCC, a lightweight object detection method based on YOLO26n. The proposed method integrates an SCC enhancement structure composed of SPDConv, CBAM, and ContextAggregation while maintaining a compact model scale. It improves feature representation and target discrimination from three complementary aspects: local detail preservation, attention-based feature refinement, and contextual semantic aggregation. Experimental results show that YOLO-SCC maintains a lightweight model scale, with 2.88M parameters and 6.0GFLOPs. Compared with YOLO26n, YOLO-SCC improves precision from 0.827 to 0.888 and mAP50-95 from 0.479 to 0.512, while recall increases from 0.813 to 0.822 and mAP50 increases from 0.852 to 0.853. These results suggest that the proposed architecture primarily improves false-positive suppression and localization quality in complex underground environments while preserving the lightweight characteristics required for practical deployment. The experimental results support the effectiveness of the proposed SCC enhancement structure under the evaluated conditions.

Furthermore, a comparative visual analysis of detection results reveals that YOLO-SCC exhibits stronger detection performance in various challenging scenarios. Compared to YOLO26n, the proposed method shows a significant reduction in missed and false detections under conditions such as low light, small target scales, and occlusion. Particularly in samples with severe local occlusion, such as miner targets where only the helmet is visible, YOLO26n often misses detections due to excessive focus on local receptive fields, whereas YOLO-SCC can effectively associate local features with the surrounding environment, achieving more accurate personnel identification. This indicates that the proposed method not only improves numerical metrics but also better adapts to the complex visual environment of underground coal mines in terms of practical detection effectiveness.

In summary, while retaining the advantages of a lightweight model, YOLO-SCC significantly enhances the comprehensive performance of underground personnel detection. Under interference from strong light and complex backgrounds, YOLO-SCC maintains excellent target perception capabilities, and the model’s focus consistently remains on the target itself. Future work will focus on evaluating YOLO-SCC on edge devices and investigating its on-device inference latency to further validate its practical deployment capability in real underground coal mine environments.

## Figures and Tables

**Table 1:** Design rationale and feature flow mechanism of the SCC enhancement structure.

Module	Name Placement	Mechanistic Role and Main Problem Addressed
SPDConv	Early downsampling layers of the backbone	Preserves local edge and texture cues before feature compression, providing more reliable detail information for subsequent attention-based filtering.
CBAM-Backbone	Middle layers of the backbone	Selectively enhances personnel-related responses based on preserved features and suppresses interference from miner lamps, equipment structures, dust, and reflections.
ContextAggregation	After feature fusion in the neck	Aggregates broader contextual information from attention-refined features to resolve ambiguity caused by occlusion, dense targets, and visually similar backgrounds.
CBAM-Neck	Before generating shallow output features	Further refines detail-rich shallow features after fusion while suppressing residual noise.

**Table 2:** Statistics of the constructed underground personnel detection dataset.

Dataset Split	Images	Annotated Personnel Instances	Average Instances per Image
Training set	2538	5277	2.079
Validation set	541	919	1.699
Test set	550	854	1.553
Total	3629	7050	1.943

**Table 3:** Experimental parameter settings.

Parameter Name	Configuration
Epochs	300
Batch	64
Worker	8
Optimizer	AdamW
Warmup_epochs	10
Weight_decay	0.001
Mixup	0.15
Mosaic	1
Close_mosaic	20

**Table 4:** Computer configuration parameters.

Hardware Name	Hardware Parameters
GPU	RTX A4000
CPU	11th Gen intel(R) core(TM)i7-11700K
Python	3.11.7
CUDA	12.1
Pytorch	2.2.1

**Table 5:** Detailed results of the ablation study.

Project	P	R	mAP50	mAP50-95	Params	GFLOPs
26n	0.827	0.813	0.852	0.479	2.375031	5.2
26n + ContextAggregation	0.909	0.781	0.856	0.481	2.292026	5.1
26n + CBAM	0.906	0.794	0.857	0.48	2.292741	5.1
26n + SPDConv	0.904	0.744	0.823	0.462	2.88079	6
26n + SPDConv + ContextAggregation	0.86	0.807	0.852	0.497	2.881442	6
26n + SPDConv + CBAM	0.854	0.747	0.82	0.482	2.882157	6
26n + ContextAggregation + CBAM	0.831	0.785	0.845	0.49	2.957744	7.3
YOLO-SCC	0.888	0.822	0.853	0.512	2.8828	6

**Table 6:** Comparative experiment results with state-of-the-art methods.

Project	P	R	mAP50	mAP50-95	Params	GFLOPs	FPS
Faster R-CNN	0.5066	0.897	0.857	0.435	137.099	370.21	17.5
RT-DETR-R50	0.83	0.764	0.806	0.466	41.936739	125.6	44.84
Rep-YOLO	0.862	0.802	0.862	0.476	23.066808	68.9	60.24
SSD	0.9181	0.7084	0.7949	0.504	26.285	62.747	61.8
v8n	0.874	0.84	0.852	0.5	3.005843	8.1	218.45
v10n	0.875	0.787	0.848	0.498	3.539271	11.1	204.28
v11n	0.865	0.821	0.845	0.505	2.58247	6.3	181.82
v12n	0.852	0.763	0.815	0.485	2.556923	6.3	161.29
v26n	0.827	0.813	0.852	0.479	2.375031	5.2	188.68
YOLO-SCC	0.888	0.822	0.853	0.512	2.8828	6	192.31

## Data Availability

Data is contained within the article.
